# Relationships between Structure and Antioxidant Capacity and Activity of Glycosylated Flavonols

**DOI:** 10.3390/foods10040849

**Published:** 2021-04-14

**Authors:** Zhengcao Xiao, Liangliang He, Xiaohui Hou, Jianping Wei, Xiaoyu Ma, Zihan Gao, Yahong Yuan, Jianbo Xiao, Pengmin Li, Tianli Yue

**Affiliations:** 1 College of Food Science and Technology, Northwest University, Xi’an 710069, China; xzc_0008@nwu.edu.cn (Z.X.); heliangliang@nwu.edu.cn (L.H.); houxiaohui@stumail.nwu.edu.cn (X.H.); jianpingwei0327@nwu.edu.cn (J.W.); maxiaoyu@stumail.nwu.edu.cn (X.M.); 13891025047@163.com (Z.G.); 2 State Key Laboratory of Crop Stress Biology for Arid Areas/Shaanxi Key Laboratory of Apple, College of Horticulture, Northwest A&F University, Yangling 712100, China; lipm@nwsuaf.edu.cn; 3 Laboratory of Nutritional and Healthy Food-Individuation Manufacturing Engineering, Xi’an 710069, China; 4 Research Center of Food Safety Risk Assessment and Control, Xi’an 710069, China; 5 College of Food Science and Engineering, Northwest A&F University, Yangling 712100, China; 2011130004@nwsuaf.edu.cm; 6 Department of Analytical Chemistry and Food Science, Faculty of Food Science and Technology, University of Vigo, 36310 Vigo, Spain; jianboxiao@uvigo.es; 7 International Research Center for Food Nutrition and Safety, Jiangsu University, Zhenjiang 212013, China

**Keywords:** flavonols, glycosylation, structure-antioxidant capacity and activity relationship, deprotonation, LC–MS

## Abstract

The antioxidant capacity (AC) and antioxidant activity (AA) of three flavonols (FLV), aglycones and their glycosylated derivatives were evaluated using 2,2-diphenyl-1-picrylhydrazyl (DPPH) and 2,2′-azino-bis(3-ethylbenzothiazoline-6-sulfonic acid) (ABTS) assays in various solvents. Findings confirmed that the glycosylation at the 3-position (3-glycosylation) always decreased the AC under most conditions due to substitution of the 3-position hydroxyl group and glycoside disruption in the molecular planarity. The 7-glycosylated derivatives did not have the above effects, thus generally exhibited ACs similar to their aglycones. Glycosylation decreased the AA of kaempferol and isorhamnetin for both assays in methanol, 3-glycosylation inhibited quercetin AA in the ABTS assay. In the DPPH assay, the AA of 3-glycosylated quercetin was significantly higher than quercetin. Using LC–MS/MS analysis, we found that quercetin and quercetin-7-glucoside underwent dimerization during the antioxidant reaction, potentially leading to a decline in AAs. However, 3-glycoside substitution may have hindered dimer formation, thereby allowing the FLVs to retain strong free radical scavenging abilities.

## 1. Introduction

Antioxidants often play dual roles in foods, added artificially to prevent the oxidation of food constituents [1,2], and as natural constituents in food benefit the human body by correcting metabolic syndrome-associated oxidative stress [3,4]. Therefore, the development and evaluation of antioxidants is an important area of research in food science. Assays using artificial free radicals, including 2,2′-azino-bis(3-ethylbenzothiazoline-6-sulfonic acid) (ABTS) and 2,2-diphenyl-1-picrylhydrazyl (DPPH), have been developed to evaluate the antioxidant potency of compounds in food [5] to standardize the measurement of nonenzymatic antioxidant ability. The two most commonly used indicators are antioxidant capacity (AC) and antioxidant activity (AA). AC evaluation requires end-point assays measuring the efficiency of antioxidant action, and the antioxidant potency is determined based on the number of free radicals scavenged by an antioxidant compound at reaction equilibrium. By contrast, AA is a kinetic-based assay that measures the reaction rate, thus reflecting the rate at which molecules scavenge free radicals [6].

Flavonols (FLVs) are a class of dietary flavonoids with strong antioxidant properties in terms of both AC and AA [7,8]. Various action mechanisms, including electron transfer (ET) and hydrogen atom transfer (HAT), are involved in free radical quenching by FLVs [6,9]. Furthermore, the phenolic hydroxyl group (-OH) proton in FLV can undergo deprotonation in ionizing solvents (e.g., water and methanol), and the generated FLV anion can then react with free radicals via the ET mechanism at a fast reaction rate. This process, known as the sequential proton-loss electron transfer (SPL-ET) mechanism, is different from the single-electron transfer followed by the proton transfer (SET-PT) mechanism that occurs without deprotonation. Because the HAT mechanism does not require deprotonation, it occurs more readily than SET-PT in nonpolar solvents [8,10]. Therefore, the dominant mechanism in free radical scavenging reactions of FLVs depends on the extent to which the antioxidant compound undergoes ionization into its conjugate base (FLV anion) [11]. These mechanisms can occur simultaneously during antioxidant-free radical scavenging by antioxidant compounds. Previous studies on FLV antioxidant mechanisms have mainly focused on the parent aglycones [7,12,13]. Limited research is available to clarify the mechanisms by which FLV glycosylated derivatives, the most common type of flavonols found in diet [14], scavenge free radicals. Typical FLVs include isoquercitrin and rutin, widely found in grains, fruits, and vegetables [15]. Quercetin-7-*O*-glucoside is found in a black seed cultivar of cowpeas [16], and isorhamnetin glycosylated derivatives are a class of flavonoids found in pears [17]. Moreover, astragalin is a glycosylated FLV widely found in nature [18]. Therefore, elucidating the antioxidation mechanism of glycosylated FLV compounds helps the research and utilization of these bioactive compounds.

**Figure 1:**
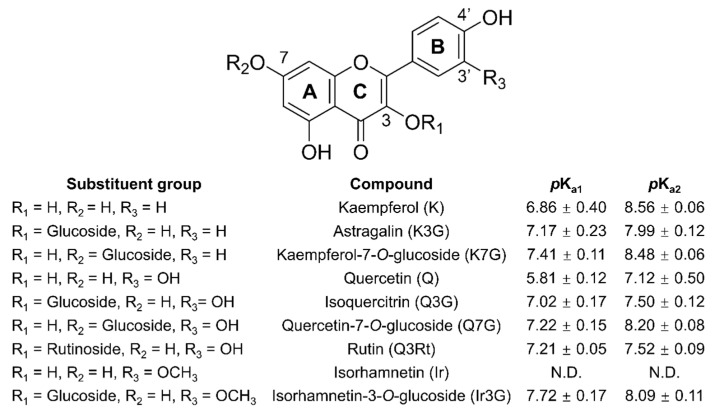
Chemical structures of flavonols and p*K*_a_ values of the compounds.

In this study, nine FLV compounds, including three aglycones and their glycosylated derivatives, were selected to investigate their antioxidant mechanism (Figure 1). The AC and AA of these compounds were evaluated using two artificial free radicals, DPPH and ABTS. We determined the dissociation constants (p*K*_a_) of the above compounds and their oxidation products to outline the antioxidant process and determine glycoside substituents’ effect on the antioxidant mechanisms of FLVs.

## 2. Materials and Methods

### 2.1. Chemicals and Reagents

Astragalin (kaempferol-3-*O*-glucoside) and isoquercitrin (quercetin-3-*O*-glucoside) were extracted and isolated from crabapple fruits (*Malus* “Winter Red”) using the reported methods, and kaempferol and quercetin were obtained by hydrolysis of afzelin and quercitrin, respectively [19]. Kaempferol-7-*O*-glucoside, quercetin-7-*O*-glucoside, rutin (quercetin-3-*O*-rutinoside), isorhamnetin, and isorhamnetin-3-*O*-glucoside were purchased from Yuanye Bio-Technology (Shanghai, China). Potassium peroxodisulfate (K_2_S_2_O_8_), phosphoric acid (H_3_PO_4_), sodium phosphate monobasic dihydrate (NaH_2_PO_4_·2H_2_O), disodium hydrogen phosphate dodecahydrate (Na_2_HPO_4_·12H_2_O), ABTS, and DPPH were purchased from J&K Scientific (Beijing, China). Ultra-pure water was prepared using a Millipore Milli-Q system (Darmstadt, Germany). All water used was ultrapure unless otherwise noted. Methanol, acetone, acetonitrile, and formic acid (LC-MS grade) were purchased from Fisher Scientific (Pittsburgh, PA, USA). All solvents were degassed with dry nitrogen to remove dissolved O_2_ and CO_2_ before use.

### 2.2. Calculation of FLV Dissociation Constants

The p*K*_a_ values for all FLVs in water were determined as described by Ramešová et al. [13] with some modifications. First, 30 μM of each FLV compound solution was briefly acidified to pH 2.5 using hydrochloric acid and then titrated with potassium hydroxide to pH 13. The titration process was monitored using a precision pH meter (PB-10, Sartorius, Goettingen, Germany) at 25 ± 0.5 °C, and the titration flask was purged using a flow of nitrogen. The p*K*_a_ determinations were completed at a variable ionic strength range of 0.01 to 0.15. Absorption spectra for FLV solutions at different pH values were obtained using a UV-2600 spectrophotometer (Shimadzu, Kyoto, Japan) with 1 cm path-length quartz cells at 25 ± 1 °C. The p*K*_a_ was calculated from the pH using the measured absorbance values according to Equation (1):

(1) $$\mathrm{pH}=pK_{a}+\log\frac{\left(A-A_{min}\right)}{\left(A_{max}-A\right)}$$

The *A_max_* and *A_min_* values represent the highest absorbance measured at the maximum and minimum pH values of the curve, respectively. Plots of log[(*A* − *A_min_*)/(*A_max_* − *A*)] against pH are linear, with the intercept equal to the p*K*_a_. All determinations were performed in triplicate. The FLV species distribution diagrams were calculated using CurTiPot [20].

### 2.3. Antioxidant Capacity Evaluation

The ABTS assay was performed according to a published method [21], with some modifications. ABTS (7 mM) solution and potassium peroxodisulfate solution (2.5 mM) were mixed and kept in the dark for 12 h to produce ABTS free radicals (ABTS^•+^). The ABTS^•+^ solutions were then diluted with water, acetone, or phosphate buffer (PBS, 50 mM) at pH s of 2.0, 4.0, and 6.0, respectively. The PBS solutions were prepared using phosphoric acid, sodium phosphate monobasic and disodium hydrogen phosphate solutions (The concentration of all solutions was 50 mM). The final absorbance of the ABTS^•+^ solution was 0.90 ± 0.05 at 734 nm, and it was used immediately. After the addition of 20 μL of 5 μM FLVs to 180 μL of diluted ABTS^•+^ solutions, the mixtures were placed in the dark for 30 min. The absorbance was then measured using an Infinite^®^ 200 Pro plate reader (Tecan, Männedorf, Switzerland) at 723 nm for the acetone mixtures and at 734 nm for all other mixtures.

The DPPH assay was performed according to the protocol developed by Sousa et al. [22] with some modifications. The DPPH (100 μM) was prepared in separate solutions containing either 40% methanol–water, acetone, or 40% methanol–PBS buffer (50 mM) at pH s of 2.5, 4.0, and 6.0, respectively, and then used for AC measurements. After adding 20 μL of 5 μM FLVs to 180 μL of the DPPH solutions, the mixtures were left in the dark for 30 min. The absorbance was measured using an Infinite^®^ 200 Pro plate reader at 517 nm for acetone mixtures and at 529 nm for all other mixtures. The pH s of all PBS buffers and methanol–PBS buffers were accurately adjusted using a pH meter. All treatments were repeated five times and conducted at 25 ± 1 °C.

The ABTS^•+^ or DPPH^•^ scavenging efficiency was used to represent the AC of compounds. The scavenging efficiency was calculated according to Equation (2):

(2) $$\mathrm{Scavenging}\;\mathrm{efficiency}\;\left(\%\right)=\frac{A_{control}-A_{sample}}{A_{control}}\times 100$$

In this equation, *A*_control_ is the absorbance of the control, which contains only free radicals and solvents. *A*_sample_ is the absorbance of the sample in the presence of test compounds.

### 2.4. Antioxidant Ability Determination

The AA of FLV was determined based on the rate constants (*k*_exp_) for the reactions between FLVs and free radicals, and *k*_exp_ values were obtained following the procedure from a previous study [23], with some modifications. FLVs (1 mM) and free radicals (DPPH^•^ and ABTS^•+^, 50 μM) were prepared separately in methanol and in acetone. Clean disposable syringes were used to individually inject the FLV and free radical solutions into a stopped-flow device (SF3, equipped with Chirascan V100, Applied Photophysics, Leatherhead, UK). The two solutions were mixed in the device, and for methanol, DPPH^•^ decay was detected at 529 nm, and ABTS^•+^ decay was detected at 734 nm. In acetone, DPPH^•^ decay was detected at 517 nm, and ABTS^•+^ decay was detected at 723 nm. Quadruplicate experiments were carried out at 25 ± 0.1 °C for all treatments. In conditions where FLV concentrations were significantly greater than free radical concentrations, the reactions of FLVs and free radicals were analyzed as pseudo-first-order processes. The *k*_exp_ was calculated using the decay plot of the free radical absorbance, following Equation (3):

(3) $$A_{t}=A_{o}exp\left(-k_{exp}t\right)+a$$

In this equation, *A_t_* is the absorbance value measured by the stopped-flow device, *t* is the reaction time, *A_o_* and *a* are the reaction constants, and *k*_exp_ (s^−1^) is the reaction rate constant.

### 2.5. Detection of Antioxidant Reaction Products

UPLC-ESI-MS was used to detect the products formed from the antioxidants during the reactions. A 1290 Infinity II UPLC system coupled to a 6470B triple-quadrupole mass spectrometer with jet stream technology electrospray ionization interface was used for mass analysis and detection (Agilent Technologies, Santa Clara, CA, USA). FLVs and free radicals (DPPH^•^ and ABTS^•+^) were diluted to 0.1 M with methanol and 1 M with acetone. The FLV compounds and free radicals with different concentrations in the same solvent were mixed separately in equal volumes. The mixtures were placed in a cooled sampler (4 °C) after filtering through 0.22 μm filters. Then, 2 μL mixtures were immediately injected into the UPLC system equipped with an Eclipse Plus column (1.8 μm, 2.1 mm × 50 mm) for separation under specific conditions. Mobile phase A consisted of 0.1% formic acid in acetonitrile, and mobile phase B consisted of 0.1% formic acid in water. The gradient used was 95% B (0 min), 70% B (4 min), 35% B (11 min), 0% B (11.5 min), 0% B (12.5 min), and 95% B (13 min) with a post-run time of 1 min, a flow rate of 0.3 mL·min^−1^, and a column temperature of 30 °C. Negative ion mass spectra were recorded in a range from 200–1000 *m*/*z*. The instrument was operated with a capillary voltage of 5000 V, nozzle voltage of 500 V, the gas temperature of 300 °C, the gas flow of 5 L·min^−1^, nebulizer of 45 psi, sheath gas flow of 11 L·min^−1^, and sheath gas heather of 250 °C. The chemical structures of characteristic oxidation products were identified using MS/MS.

### 2.6. Statistical Analysis

Significant differences were detected by *t*-tests using SPSS 16.0 software (IBM, New York, NY, USA), with *p* < 0.05 considered significant.

## 3. Results and Discussion

### 3.1. Antioxidant Capacity and Ability of FLVs

**Figure 2:**
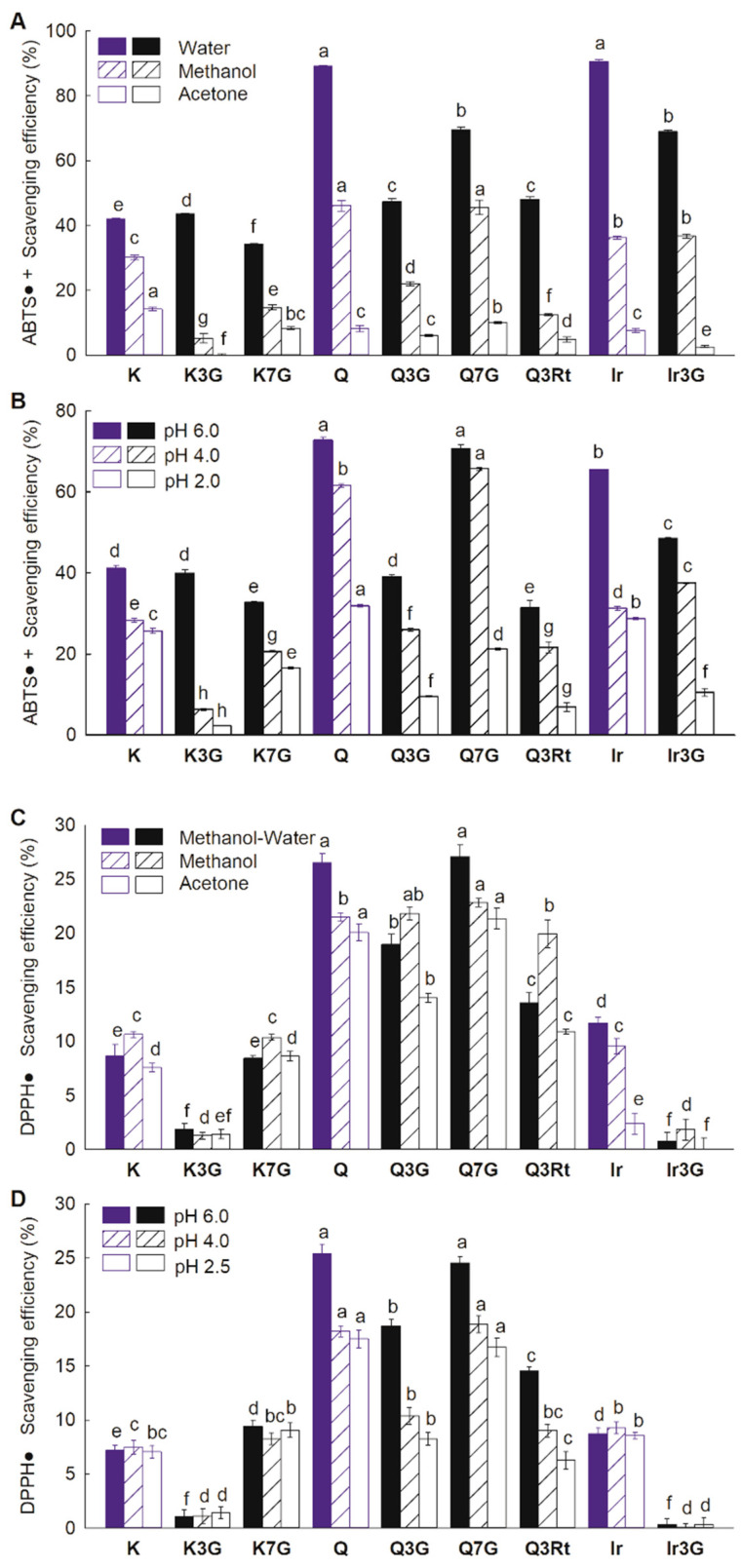
Antioxidant capacity of flavonols evaluated using 2,2′-azino-bis(3-ethylbenzothiazoline-6-sulfonic acid) (ABTS) assay in different solvents (**A**) or in phosphate buffer (PBS) buffers with different pH (**B**) and evaluated using 2,2-diphenyl-1-picrylhydrazyl (DPPH) assays in different solvents (**C**) or in PBS buffers (**D**). Data are shown as mean ± SE (*n* = 5). Names of the flavonol compounds are presented in Figure 1. The different small letters indicate significant differences (*p* < 0.05) among compounds in the same solution or condition.

The ACs of all compounds, specifically kaempferol (K), kaempferol-3-*O*-glucoside (K3G), kaempferol-7-*O*-glucoside (K7G), quercetin (Q), quercetin-3-*O*-glucoside (Q3G), quercetin-3-*O*-rutinoside (Q3Rt), quercetin-7-*O*-glucoside (Q7G), isorhamnetin (Ir), and isorhamnetin-3-*O*-glucoside (Ir3G), were evaluated using ABTS and DPPH assays (Figure 2). For the ABTS assay, two aglycones, Q and Ir, had the highest ACs in water. All glycosylated derivatives except K3G had lower ACs than their aglycones in water. The ACs of FLV measured in methanol were lower than those measured in water, and AC of K3G was significantly lower than that of its aglycone. Meanwhile, Q7G and Ir3G ACs were equal to their respective aglycones, but the ACs of other glycosylated derivatives were lower than their aglycones. When the reaction solvent was changed to acetone, a further reduction in the compounds’ ACs was observed, with the ACs of all 3-glycosylated derivatives lower than those of their aglycones. For the 7-glycosylated derivatives, the ACs of K7G and Q7G were lower and higher than their aglycones, respectively. A decrease in the ACs for all compounds was also observed as the reaction buffer acidity increased (Figure 2B). The AC profiles measured in the buffer solutions were similar to those measured in different solvents (Figure 2). However, there were some relevant differences between the results, the order of the aglycone AC being determined to be in the order Q > Ir > K. K3G showed a lower AC than K in the pH 2 and pH 4 buffers, while Q7G showed a higher AC than Q in the pH 4 buffer. Significantly, at pH 2, the AC decreased to a greater extent with 3-glycosylation than with 7-glycosylation.

For the DPPH assay, the ACs of the compounds Q, Q7G, and Ir were decreased by changing the reaction solvents from water to organics. The Q3G and Q3Rt showed higher ACs than those in the methanol–water mixture (Figure 2C). We speculate that methanol–water as the reaction solvent may weaken the reaction of the hydrophobic DPPH^•^ and some hydrophilic FLV (like 3-glycosylated FLV). Among the three aglycones, Q had the highest AC in all solvents, and K and Ir had the lowest ACs in methanol–water and acetone, respectively. The ACs of K and Ir were the same in methanol. The ACs of the 3-glycosylated FLVs were lower than the corresponding aglycones, while the ACs of 7-glycosylated derivatives were equal to or greater than the aglycones in the three solvents. Reaction buffers with increased acidity only caused a decreased AC of Q and its glycosylated derivatives (Figure 2D). The aglycone AC order was the same in all buffers compared to those measured in methanol–water and methanol. In addition, the effects of glycosylation were similar to the ABTS assay, except that 3-glycosylation strongly inhibited the AC of K, while 7-glycosylation marginally promoted it.

**Figure 3:**
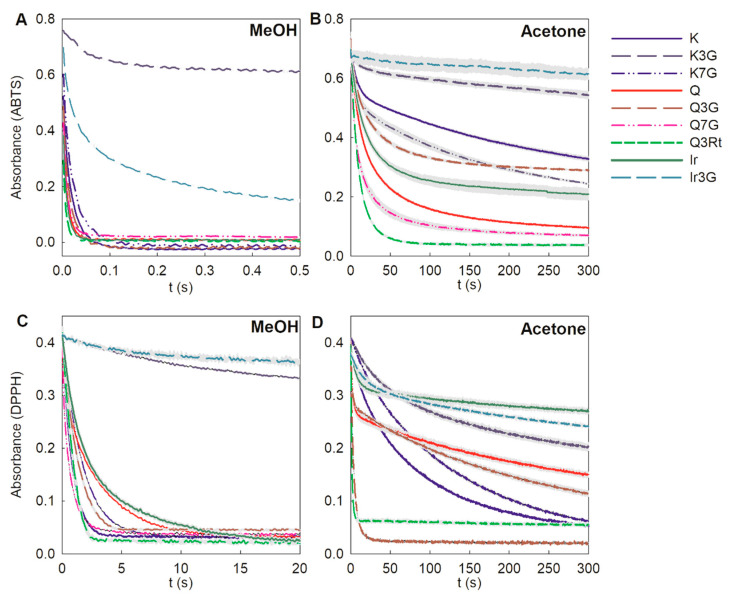
Decay curves of ABTS^•+^ (**A**,**B**) and DPPH• (**C**,**D**) when quenched by flavonols in methanol and acetone, respectively. Each curve was obtained by taking the average of five curves, and the gray area represents the SE of each curve. Names of the dihydrochalcone compounds are presented in Figure 1.

AC is used to calculate quantities of free radicals scavenged per antioxidant, while AA is used to determine the speed at which free radicals are scavenged. The AAs of all compounds for ABTS^•+^ and DPPH^•^ were evaluated using the stopped-flow device. However, since the reaction rate of FLVs with ABTS^•+^ in methanol is too high, part of the reaction, in which the period is less than the stopped-flow instrument dead time (2.5 × 10^−4^ s), cannot be recorded (Figure 3A). Thus, we approximated the fit for the decay curves of ABTS^•+^ absorbance to allow the indirect comparison of the AAs. Moreover, the absorbance at 2.5 × 10^−4^ s can also be used to compare the AAs of these compounds (Supporting Information supplementary Figure S1).

**Figure 4:**
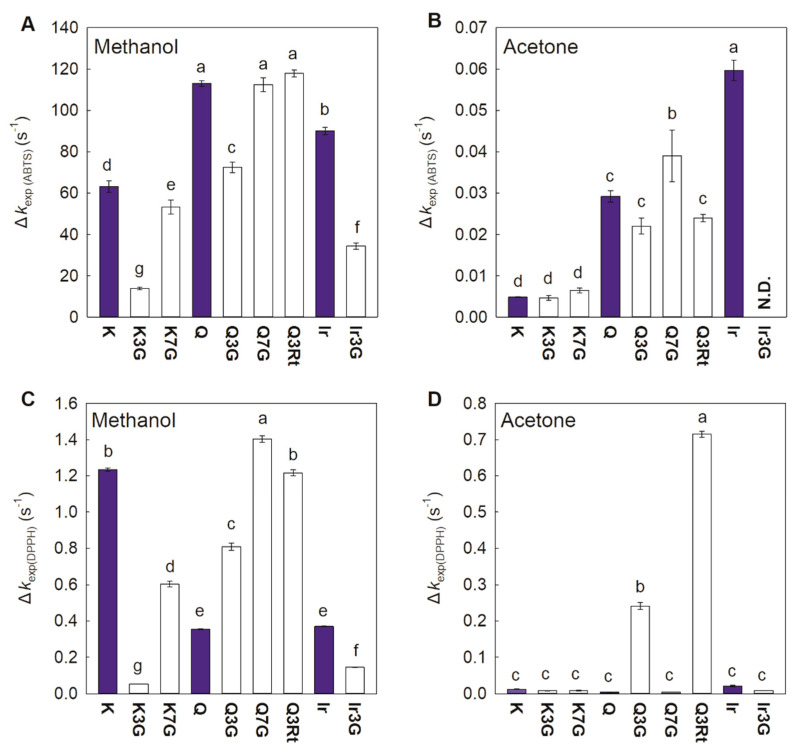
Antioxidant activity of flavonols evaluated using ABTS (**A**,**B**) and DPPH (**C**,**D**) assays in methanol and acetone. Data are shown as mean ± SE (*n* = 5). Names of the flavonol compounds are presented in Figure 1. Different small letters indicate significant differences in the same condition (*p* < 0.05).

Among the three aglycones that reacted with ABTS^•+^ in methanol, Q had the highest AA, while K had the lowest (Figure 4A). K3G, K7G, Q3G, and Ir3G had lower AAs than their aglycones, and the AA of K7G was higher than that of K3G. Two other Q derivatives, Q3Rt and Q7G, exhibited similar AAs to their aglycones. Switching from the protic solvent methanol to the aprotic solvent acetone decreased the *k*_exp_ values of FLVs reacted with ABTS^•+^, with AAs of the three aglycones ordered as follows: Ir > Q > K (Figure 3B). For the derivatives, glycosylation did not greatly influence the AA of K, whereas only a small but non-significant decrease in the AA of Q was observed for 3-glycosylation, and an increase in the AA was observed for 7-glycosylation. The *k*_exp_ of Ir3G was too small to be measured. The *k*_exp_ of FLVs measured in methanol was smaller for DPPH^•^ than it was for ABTS^•+^ (Figure 3C). The highest AA among the three aglycones was observed with K, and the AAs for other aglycones were the same. For the derivatives, 3-glycosylation caused a greater decrease in AA than 7-glycosylation for K, and it also decreased the AA for Ir. However, glycosylation enhanced the AA for Q, especially at the 7-position, and rutinoside caused a greater increase than glucoside in AA at the 3-position. The AAs of all compounds were lower in acetone than in methanol for the DPPH assay, similarly to the ABTS assay (Figure 3D). Both 3-glycosylated derivatives of Q, in particular Q3Rt, showed high AAs than other compounds. Moreover, the *k*_exp_ values of these two compounds were so high that there was no significant difference when statistically analyzing the *k*_exp_ values of all FLVs together.

The extent to which the glycosylation of FLVs affected AC and AA depended on aglycone chemical structure, reaction conditions, and the nature of the free radicals being employed. Therefore, the antioxidant potency of the FLV aglycones should not be used to infer the antioxidant property of the corresponding glycosylated derivatives.

### 3.2. Antioxidant Mechanism of Glycosylated FLVs

Antioxidant molecules donate electrons or hydrogen atoms to quench free radicals [10]. Therefore, AC can be understood in terms of the number of electrons or hydrogen atoms donated by an antioxidant molecule to neutralize free radicals for an antioxidant molecule because AC measurements are conducted with an excess of free radicals. Antioxidant compounds with several -OHs exhibit higher ACs through deprotonation due to the formation of anions with a greater capacity than the corresponding neutral molecules to quench free radicals [11]. Therefore, the deprotonation abilities of those FLV compounds were determined in aqueous solutions, except for Ir, which is insoluble in aqueous media. Glycosylation decreases the extent of deprotonation, and therefore, increases the p*K*_a_ values (Figure 1). Greater conjugation can stabilize anions and is, therefore, beneficial to ionization. However, the steric hindrance of 3-glycoside forces the B-ring not to be coplanar with the A- and C-ring, which weakens the conjugation system of the entire molecule [24]. Based on the above analysis, Ir is expected to have a lower p*K*_a_ value than Ir3G. The 7-OH usually deprotonates when the compounds dissolve in ionizing solvents [25]. Thus, the 7-OH substitution of FLV decreased the deprotonation ability.

According to the distribution diagrams of these compounds (Supporting Information supplementary Figure S2), it can be considered that only the first deprotonation takes place in aqueous solutions and pH 6 buffers. Due to SPLET mechanism advantages in kinetics [8], the AC measured in aqueous solutions and pH 6 buffers are mainly dependent on FLV anions with a single negative charge. Moreover, in the nonionizing solvent acetone, or in buffers with pH 2, the ACs represent the neutral molecules, whereas the ACs measured in pure methanol and pH 4 buffers represent a transition between the anion and neutral molecule. Several factors explain the decrease in the AC of 3-glycosylation for most aglycones, whether in neutral or ionized form. Reportedly, 3-OH plays a key role in compounds with high ACs [6]; therefore, the substitution of this group leads to a decreased AC of all FLV compounds. Moreover, 3-glycoside with steric hindrance prevents coplanarity between the A-, B-, and C-rings, thereby reducing π electron delocalization in the aromatic ring system and thus decreasing the AC [24]. In addition, the reaction between FLV and free radicals is hindered by the increased steric hindrance induced in the 3-glycoside derivatives. The 7-glycosylation does not have the above factors; therefore, there were fewer effects from the 7-glycosylated substitution.

The ACs of the K3G anion and the K7G anion measured by the ABTS assay were equal to or lower than that of the K anion, respectively, indicating that FLV anions could quench the ABTS^•+^ through the 7-OH group at the A-ring instead of the 3-OH group when the molecule is missing the *o*-dihydroxyl group in the B-ring. However, neutral molecules only can quench ABTS^•+^ using 3-OH. For example, the AC of K3G was lower than that of K in acetone and pH 2 buffer. Moreover, in compound K, the first ionization occurs at 7-OH [25]. Therefore, glycosylated 7-OH decreases the level of ionization, further affecting the AC of anions. In addition, ABTS^•+^ is more active than DPPH^•^ [26], so it may be able to abstract electrons from the K3G anion where DPPH^•^ cannot. Therefore, the K3G anion has lower AC for DPPH^•^ than the K anion or the K7G anion.

AA can be understood as the rate at which electrons or hydrogen atoms can be transferred from antioxidant molecules to free radicals. It is widely recognized that kinetic mechanisms depend on several thermochemical parameters of antioxidant compounds, including bond dissociation enthalpy, ionization potential, and electron transfer enthalpy [27,28]. However, previously reported theoretical data could not explain the FLV AA results. First, ABTS and DPPH assays provide different AA values, both of which cannot simultaneously explain by one theoretical result. Moreover, the parameters of the theoretical studies are inconsistent with each other (as detailed in Supporting Information supplementary Table S1). Therefore, we attempt to explain the results using the molecular structure of FLV.

Due to the ease with which ABTS^•+^ is quenched through electron transfer [26], it can be compared the ABTS^•+^ scavenging activities via the ET mechanism of FLV neutral molecules using the results in acetone (Figure 4B). The FLVs with *o*-dihydroxyl groups and methylated *o*-dihydroxyl groups had higher AAs, possibly due to the increased electron delocalization of the aromatic systems afforded by these groups. The lack of AA for Ir3G indicated the crucial role of the 3-OH group as an electron donor for quenching free radicals in Ir. When comparing Q and its derivatives, 3-glycosylation slightly decreased the AA due to the substitution of 3-OH and the changing of molecular planarity by glycosylation. The higher AA of Q7G than its aglycone suggests that 7-glycoside can improve the ability of neutral FLV molecules to donate electrons. Moreover, glycosylation had little effect on the AA of K and its derivatives, indicating that glycosylation did not affect the electron contribution ability in neutral FLV molecules with a single-OH group at the B-ring.

The AAs determined using ABTS^•+^ in methanol can be used to compare the free radical scavenging abilities of FLV anions via the ET mechanism (Figure 4A) because methanol as an ionizing solvent partly supports compound deprotonation [23,29]. As expected, AAs measured in methanol were higher than those in acetone, demonstrating a stronger electron-donating -ability of the FLV anions than the neutral molecules. Similar to the result observed in acetone, the *o*-dihydroxyl group and the methylated *o*-dihydroxyl group at the B-ring increased the AAs through electron delocalization enhancements of the FLV anion conjugated systems. Concerning FLVs bearing mono-glucoside substituents, 3-glycoside decreases the AA by replacing the 3-OH and affecting the anion planarity. Conversely, 7-glycosylation did not disturb the 3-OH or change the anion planarity, so the glycosylation substituent influence on the FLV-anion AA was smaller. In particular, Q3Rt, which contains a di-glycoside substituent group at the 3-position, exhibited a high AA for the reaction of ABTS^•+^ in methanol, despite having the rutinoside substitute for the 3-OH and a greater steric hindrance than the mono-glycoside. The confusing result may due to the complex and changeful conformation of rutinoside [30], which results in an unexpected impact on its reactivity as an antioxidant.

The AAs measured in methanol using the DPPH assay were significantly lower than those measured using the ABTS assay (Figure 4A,C), demonstrating the weaker ability of DPPH^•^ to abstract electrons from FLV anions than ABTS^•+^. Thus, multiple mechanisms are likely occurring in parallel in the DPPH^•^ scavenging process because DPPH^•^ preferentially acts through the HAT mechanism but can also be quenched via the SPLET mechanism in ionizing solvents [23,26,29,31]. Similar to the results obtained using the ABTS assay, glycosylation decreased the AA of K and Ir, possibly due to the weakening of conjugated systems delocalization or the substitution of specific -OH. Thus, far, still unexplained are the more active DPPH^•^ scavenging abilities of all glycosylated derivatives of Q than their aglycones in methanol and for the much higher AAs of Q3G and Q3Rt in acetone. Thus, LC–MS analysis was used to examine the reaction products formed during the process and to provide further insights into the antioxidant reaction mechanisms.

### 3.3. The Effect of FLV Antioxidant Products on Antioxidant Ability

**Figure 5:**
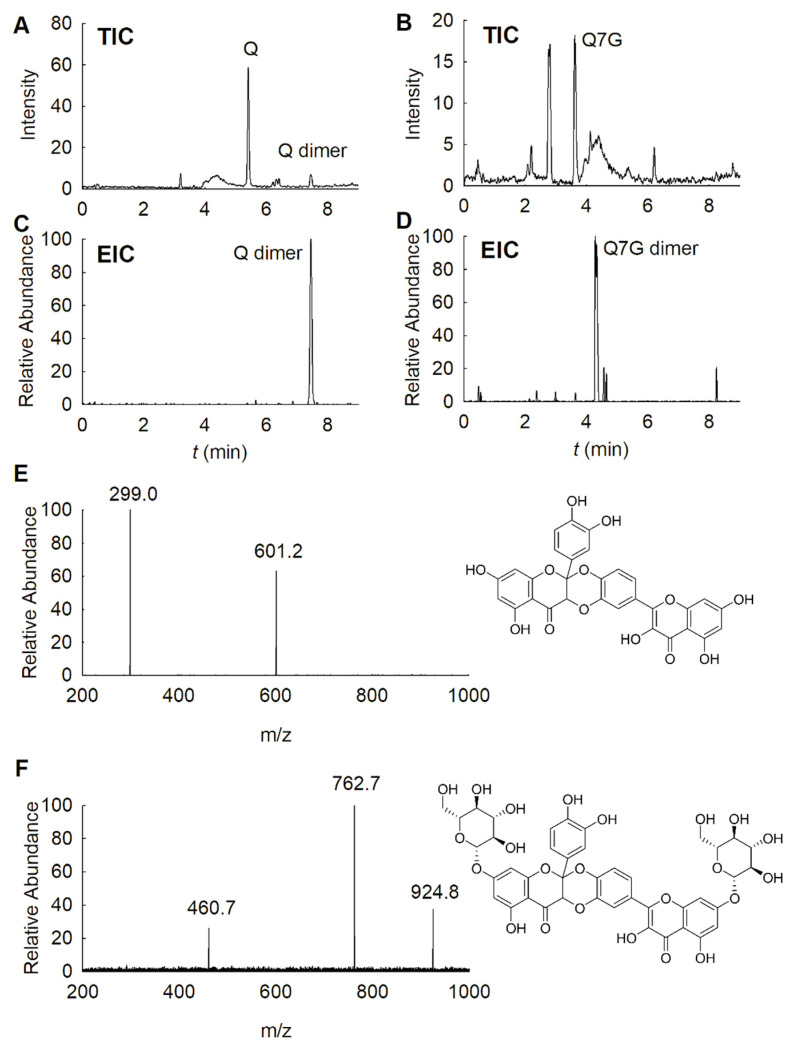
The LC–MS result of quercetin (Q) and quercetin-7-*O*-glucoside (Q7G) reacted with DPPH^•^ in acetone. Total ion chromatography (TIC) of the reactions between Q and DPPH^•^ (**A**) as well as Q7G and DPPH^•^ (**B**) in acetone. The extracted ion chromatography (EIC) (**C**,**D**) and MS/MS spectra in the negative mode and inference chemical structures of the dimers are shown (**E**,**F**).

All FLVs were mixed with the free radicals in methanol and acetone. Then they were immediately subjected to LC–MS. After reviewing the antioxidant product retention times and the fragment ions, it was found that changing the FLV and free radical concentrations had no effect on the nature of the reaction products. Different FLVs or free radicals in different solvents would be expected to afford different products. An exception is that the products of Q reacted with ABTS^•+^ in methanol and acetone were completely consistent (Supporting Information supplementary Table S2). Particularly, the dimeric products were detected in the reaction mixture of Q and Q7G, respectively reacted with DPPH^•^ in acetone (Figure 5).

The MS/MS spectra showed that the quasi-molecular ion of the dimeric product from Q was 601.2 *m*/*z*, with only one fragment ion at 299.0 *m*/*z*. Considering the MW of Q (302.24), the result indicated that the dimer was one of a quercetin Diels–Alder dimer (Figure 5E) [32]. A likely pathway for the dimer formation is one quercetin losing H atoms and being converted to an *o*-quinone, which gives a Diels–Alder reaction with another quercetin to form the dimer with an MW of 602. Similarly, a Diels–Alder dimer with an MW of 924.8 was found from the reaction of Q7G with DPPH^•^ in acetone. According to the MS/MS spectra (Figure 5F), two fragments, one at 762.7 *m*/*z* and the other at 460.7 *m*/*z*, were found. Considering the MWs of glucoside (162.16) and Q7G (464.38), the fragment with 762.7 *m*/*z* corresponds to the quasi-molecular ion of the dimer after glucoside loss, while the fragment at 460.7 *m*/*z* may be a Q7G *o*-quinone fragment. In addition, higher collision energy (>10 V) in the MS instrument leads to the disappearance of the 762.7 *m*/*z* fragments, leaving only the 460.7 *m*/*z* fragments (Supporting Information supplementary Figure S3). Although FLVs can spontaneously form dimers, it mainly occurs in PBS buffers rather than in organic solvents [33]. Therefore, the dimers apparently formed during the antioxidant reaction. The Diels–Alder reaction requires the *o*-dihydroxyl group at the B-ring and a double bond of the C-ring. Thus, no dimers were detected in the reactions of K, Ir, and their derivatives due to the absence of the *o*-dihydroxyl group. The Q-3-glycosylated derivatives could not dimerize due to the 3-glycoside hindrance. Moreover, the dimer only formed under reaction conditions favorable for the HAT mechanism (reacted with DPPH^•^ in acetone), indicating that the loss of the hydrogen atom triggered dimerization. Thus, according to the DPPH assay in acetone, a correlation may exist between the very high AAs of Q-3-glycosylated derivates and the dimer formations of Q and Q7G.

Inference was made that FLVs with the *o*-dihydroxyl group at the B-ring can dimerize with other FLVs when they lose hydrogen atoms during the reaction with DPPH^•^ via the HAT mechanism. The dimer has greater steric hindrance than the monomer FLV, and the DPPH^•^ is sensitive to steric hindrance [34], causing the inactivation of the HAT process. However, 3-glycoside in the FLV molecule blocks dimerization and thereby retains a high *k*_exp_ value in the HAT mechanism. Our hypothesis is supported by two results: first, the absorbance decay curves of DPPH^•^ scavenged by Q and Q7G have early steep slopes (<5 s), and then the curves become flatter (Figure 3D). It indicated that the reaction has a high rate at the beginning, and the rate would decrease instead of complete the reaction with a high rate like Q3G and Q3Rt. When only fit the steep curves, larger *k*_exp_ values of Q (0.459 ± 0.046 s^−1^) and Q7G (0.670 ± 0.019 s^−1^) were obtained, indicating that when Q and Q7G reacted with DPPH^•^ in acetone, they went through two stages that had different *k*_exp_ values. The high *k*_exp_ may be due to the molecules quenching the free radicals by losing a hydrogen atom from the *o*-dihydroxyl group. Meanwhile, the residues that lost hydrogen atoms immediately reacted with other FLVs to form dimers, greatly increasing the steric hindrance of the entire molecule and reducing the *k*_exp_ in the HAT reaction. Moreover, an antioxidant product with an MW of 608 (corresponding to the 607.0 *m*/*z* fragments) was found in the reaction between Q3Rt and DPPH^•^ (Supporting Information supplementary Table S2). The oxidation products with molecular weights of two units less than Q3Rt were found, indicating the ease with which Q3Rt molecules lost hydrogen atoms in the scavenging reaction with DPPH^•^. This result shows that FLV compounds with high glycosidic hindrance are more likely to quench free radicals by losing hydrogen atoms and avoid dimerization, and it is consistent with the result that Q3Rt scavenges DPPH^•^ with high AA in acetone.

We proposed a mechanism to explain the extremely high AAs of Q-3-glycosylated derivatives. According to our findings, Q-3-glycosylated derivatives may have greater reactivity than their counterpart aglycones in quenching free radicals under conditions inhibiting deprotonation, such as in acidic solutions or nonionizing solvents. Therefore, these glycosylated derivatives, including isoquercitrin, quercitrin, and rutin, may be more reactive than quercetin in scavenging small amounts of free radicals in acidic conditions.

## 4. Conclusions

In conclusion, the antioxidant evaluation of FLVs showed that 3-glycosylation generally decreased the AC of FLVs, while 7-glycosylation had a lesser effect on the AC. This result occurred due to the 3-glycoside substitute for 3-OH as well as weakened the conjugation system of FLV by affecting the planarity of the anion or neutral molecules. Under non-ionizing conditions, 3-glycosylated FLVs with the *o*-dihydroxyl group in the B-ring exhibited very high AA than their aglycones, possibly due to the hindrance of the dimerization of FLVs during the free-radical quenching process via the HAT mechanism, which inhibited the scavenging activity of the antioxidants. Thus, the presence of the 3-glycoside in FLV with the *o*-dihydroxyl group blocking the FLV dimerization helped maintain the *k*_exp_ of the reaction. Glycosylation can improve the water solubility of flavonoids, so glycosylated FLVs have wider applications than the aglycones in nonenzymatic antioxidation. Moreover, due to the complex mechanism by which glycosylation affects the antioxidant process, we suggest that antioxidant potency should be considered from the standpoint of both antioxidant capacities and antioxidant abilities.

## Data Availability

Not applicable.

## Supplementary Materials

The following are available online at https://www.mdpi.com/article/10.3390/foods10040849/s1, Figure S1: Decay curves of ABTS^•+^ in methanol when *t* < 0.05 s; Figure S2: Species distribution diagram of the FLVs; Figure S3: MS/MS spectra in the negative mode for the dimers that formed in the DPPH• scavenging reaction by Q7G in acetone under different CE; Table S1: Thermochemistry parameters included bond dissociation enthalpy (BDE), ionization potential (IP), proton dissociation enthalpy (PDE), and electron transfer enthalpy (ETE) of the flavonols; Table S2: Fragments of antioxidant products. MO, methanol; AT, acetone.
