# Supplementary material for: Relationships between Structure and Antioxidant Capacity and Activity of Glycosylated Flavonols
Source: Foods. 2021 Apr 14;10(4):849. doi: 10.3390/foods10040849 (PMC8070355; doi:10.3390/foods10040849)
Supplement: Supplementary file 1 [file foods-10-00849-s001.pdf]

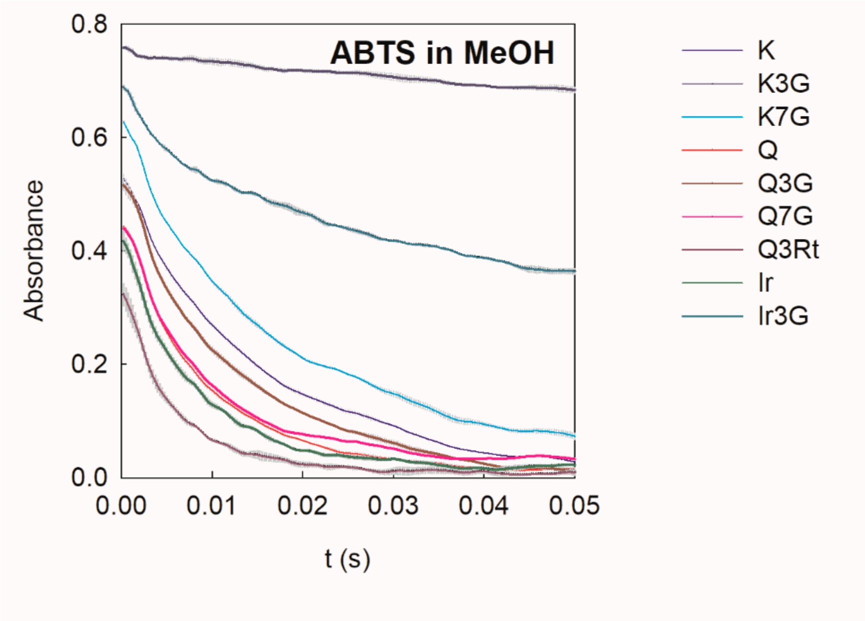

Fig. S1 Decay curves of $ABTS^{\bullet+}$ in methanol: $t < 0.05$ s.

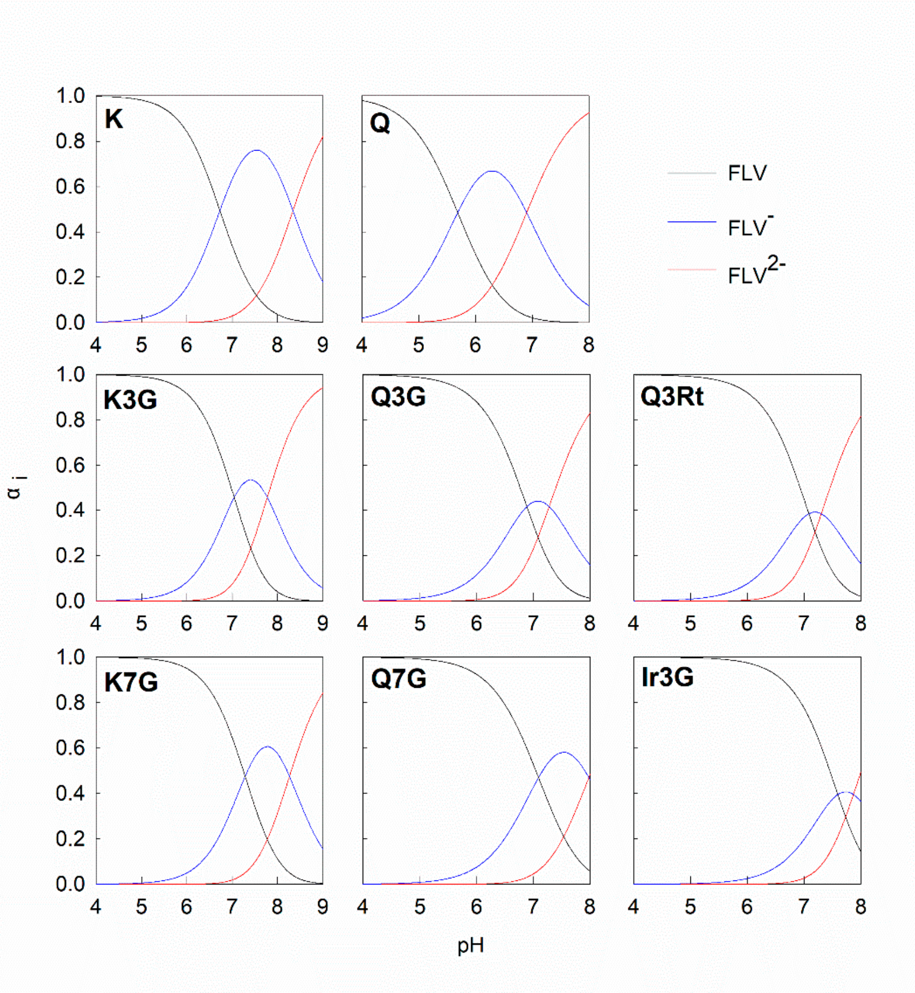

Fig. S2 Species distribution diagram for K, K3G, K7G, Q, Q3G, Q7G, Q3Rt, and Ir3G.

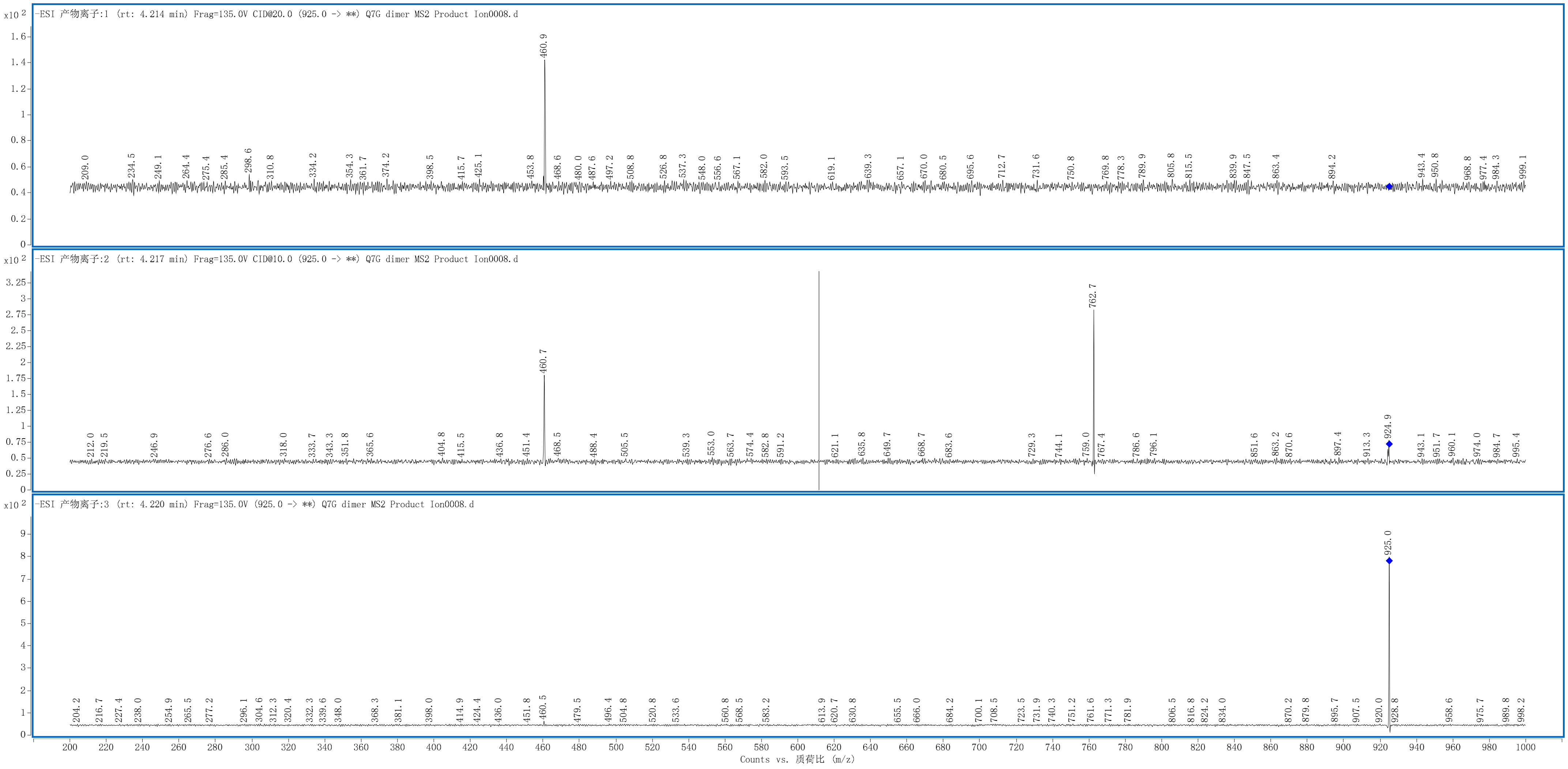

Fig. S3 MS/MS spectra in negative mode for the dimers that formed in the DPPH• scavenging reaction by Q7G in acetone under different CE. Top spectrum mean CE = 20 V, middle spectrum CE = 10 V, bottom spectrum CE = 0 V.

Table S1. Thermochemistry parameters included bond dissociation enthalpy (BDE), ionization potential (IP), proton dissociation enthalpy (PDE), and electron transfer enthalpy (ETE) of the flavonols.

		K	K3G	K7G	Q	Q3G	Q7G	Q3Rt	Ir	Ir3G
BDE	kJ/mol				343.2[a]	349.4[a]	343.6[a]			
	kJ/mol	291[b]			294.4[b]					
	kcal/mol	80.9 (73.9)[c]	83.9 (81.4)[c]		74.7 (70.9)[c]	76.6 (75.2)[c]				
	kcal/mol	86.8 (74.1)[d]			78.6 (73.7)[d]					
	kJ/mol	334.362[e]			299.284[e]	309.1[e]			310.112[e]	
	kcal/mol	394.8[f]			391.2[f]					
IP	kJ/mol				536.1[a]	556.7[a]	537[a]	N. D.		N. D.
	kJ/mol	359.9[b]			355.9[b]					
	kcal/mol	128.3[c]	138.3[c]		127[c]	136[c]				
	kcal/mol	7.08 (2.73)[d]			7.03 (2.69)[d]					
	kJ/mol	557.685[e]			553.826[e]	579.854[e]			554.79[e]	
	kcal/mol	131.8[f]			130.8[f]					
PDE	kJ/mol				-10.9[a]	-24.3[a]	-10.5[a]			
	kJ/mol	-38.3[b]			-30.9[b]					
	kJ/mol									
ETE	kJ/mol				403.7[a]	407.1[a]	403.7[a]			
	kJ/mol	289.4[b]			295.5[b]					
	kcal/mol	114[c]	116.2[c]		113[c]	114[c]				

a Zheng, Y. Z., Deng, G., Liang, Q., Chen, D. F., Guo, R., & Lai, R. C. (2017). Antioxidant Activity of Quercetin and Its Glucosides from Propolis: A Theoretical Study. Scientific Reports, 7, 11.

b Stepanic, V., Troselj, K. G., Lucic, B., Markovic, Z., & Amic, D. (2013). Bond dissociation free energy as a general parameter for flavonoid radical scavenging activity. Food chemistry, 141(2), 1562-1570.

c Lespade, L., & Bercion, S. (2012). Theoretical investigation of the effect of sugar substitution on the antioxidant properties of flavonoids. Free Radical Research, 46(3), 346-358.

d Lemanska, K., Szymusiak, H., Tyrakowska, B., Zielinski, R., Soffers, A., & Rietjens, I. (2001). The influence of pH on antioxidant properties and the mechanism of antioxidant action of hydroxyflavones. Free radical biology and medicine, 31(7), 869-881.

e Zhang, D., Chu, L., Liu, Y. X., Wang, A. L., Ji, B. P., Wu, W., … Jia, G. (2011). Analysis of the Antioxidant Capacities of Flavonoids under Different Spectrophotometric Assays Using Cyclic Voltammetry and Density Functional Theory. Journal of agricultural and food chemistry, 59(18), 10277-10285.

f Nakanishi, I., Ohkubo, K., Shoji, Y., Fujitaka, Y., Shimoda, K., Matsumoto, K.I., … Hamada, H. (2020). Relationship between the radical-scavenging activity of selected flavonols and thermodynamic parameters calculated by density functional theory. Free Radical Research, 54(7), 535-539.

Table S2. Fragments of antioxidant products. MO, methanol; AT, acetone.

	Rt (min)	*m/z*	Free radicals	Solvents	Antioxidant products									
					Rt (min)	Fragment (*m/z*)	Rt (min)	Fragment (*m/z*)	Rt (min)	Fragment (*m/z*)	Rt (min)	Fragment (*m/z*)	Rt (min)	Fragment (*m/z*)
K	6.30	301.1	DPPH	MO	4.20	315.0, 333.1	5.42	315.0, 347.1	6.80	587.0				
				AT	3.90	273.1, 301.1	4.20	315.0, 333.1	5.41	315.1, 347.1				
			ABTS	MO	3.90	273.1, 301.1	4.20	315.0, 333.1	5.41	315.1, 347.1				
				AT	2.34	258.0	3.90	273.1, 301.1						
K3G	4.04	447.2	DPPH	MO	N. D.									
				AT	3.70	463.1	6.20	514.2, 595.0						
			ABTS	MO	2.95	227.0, 255.9								
				AT	2.36	258.0	2.95	227.0, 255.9	3.85	326.0				
K7G	4.13	447.2	DPPH	MO	2.59	314.9, 495.0, 631.3	3.30	315.1, 509.4, 577.4						
				AT	2.81	301.1, 463.3								
			ABTS	MO	2.46	315.0, 333.2, 495.3	2.58	315.0, 333.2, 495.3, 563.3	2.80	301.0, 463.2				
				AT	2.36	258.0	2.80	301.0, 463.2	3.85	326.0				
Q	5.41	301.1	DPPH	MO	3.61	331.1, 349.2	4.62	331.1, 363.1	6.21	514.3	6.33	285.2	6.42	315.0
				AT	3.20	317.1, 339.0	6.21	514.3	6.33	285.2	6.42	315.0	7.45	601.0
			ABTS	MO	3.65	331.1, 349.1	4.62	331.1, 363.1	6.33	285.2	6.42	315.0		
				AT	3.65	331.1, 349.1	4.62	331.1, 363.1	6.33	285.2	6.42	315.0		
Q3G	3.72	463.1	DPPH	MO	3.61	461.1, 437.1	6.22	514.2, 587.0						
				AT	2.17	479.20	3.53	461.0, 437.1	6.22	514.2, 587.0				
			ABTS	MO	2.93	226.9, 241.4,								

						256.0								
				AT	2.36	258.00	2.92	226.9, 241.4, 256.0						
Q7G	3.51	463.1	DPPH	MO	2.10	511.1, 579.0	2.79	331.0, 493.1, 525.1, 593.1	6.21	437.1, 514.1				
				AT	2.17	479.1	4.34	461.2, 925.2	6.21	437.1, 514.1				
			ABTS	MO	2.09	511.1, 579.0	2.20	511.4, 579.4	2.78	331.1, 493.4, 525.4, 593.3	2.83	331.1, 493.4, 525.4, 593.4	2.93	255.8, 304.8
				AT	2.17	479.1	2.36	258.0	2.93	256.2, 304.9				
Q3Rt	3.51	609.1	DPPH	MO	3.30	607.0	3.83	593.1	3.88	623.2	6.21	437.1, 514.1		
				AT	3.30	607.0								
			ABTS	MO	2.92	226.9, 241.6, 255.9								
				AT	2.34	257.90	2.92	226.9, 241.6, 255.9						
Ir	6.41	315.0	DPPH	MO	4.28	345.0, 363.0	5.22	407.1, 345.0	5.42	377.1, 445.1				
				AT	3.97	331.0	4.28	345.0, 363.0	5.42	277.9, 301.0, 377.0	5.99	343.0	6.12	312.9, 331.0
			ABTS	MO	3.97	331.0	4.28	345.0, 363.0	5.19	407.1	5.39	345.1, 377.0	6.23	345.1
				AT	2.34	257.9	3.97	331.0	5.40	301.0	6.12	312.9, 331.0	6.23	345.1
Ir3G	4.07	477.2	DPPH	MO	6.20	437.1, 514.2, 587.0								
				AT	6.20	437.1, 514.2, 587.0								
			ABTS	MO	2.92	227.0, 256.1	5.39	377.00						
				AT	2.36	257.90	2.92	227.0, 256.1	3.85	326.0				
